# Evaluation of the Salivary Expression of Type I Collagen, Osteocalcin, and Osteonectin in Patients Treated With Myofunctional Therapy: A Clinical Study

**DOI:** 10.7759/cureus.89762

**Published:** 2025-08-10

**Authors:** Aishwarya Dixith S, Chaitra K R, Tarulatha R Shyagali, Vinay K, Smitha V Shetty, Sindhu D, Veekshith M, Sukrutha R

**Affiliations:** 1 Department of Orthodontics, Mathrusri Ramabai Ambedkar Dental College and Hospital, Bangalore, IND

**Keywords:** bone remodelling, class ii malocclusion, collagen type i, mandibular retrognathism, myofunctional therapy, orthodontics, osteocalcin, osteonectin, salivary biomarkers, twin block appliance

## Abstract

Introduction

Skeletal Class II malocclusion, commonly resulting from mandibular retrognathism, poses both functional and aesthetic challenges in growing patients. Functional orthopaedic appliances, such as the Twin Block, aim to correct this by stimulating mandibular growth through forward positioning. This biomechanical stimulus is believed to trigger adaptive remodelling at the condylar cartilage level, influencing bone formation pathways and playing a crucial role in the release of salivary biomarkers such as osteocalcin, osteonectin, and collagen type 1, which can be non-invasively detected.

Aim

This study aims to assess the salivary levels of collagen type I, osteocalcin, and osteonectin in skeletal Class II patients undergoing myofunctional therapy with a Twin Block appliance.

Methods

A total of 10 adolescents aged 8-15 years with skeletal Class II malocclusion were selected and treated with Twin Block appliances. Unstimulated saliva samples were collected at four time points: baseline (prior to appliance insertion) and 15, 30, and 60 days post-insertion. Samples were stored at -82°C and analysed for type I collagen, osteocalcin, and osteonectin using biochemical assays.

Results

All three biomarkers (collagen type I, osteocalcin, and osteonectin) showed a consistent pattern of expression. Levels increased from baseline and peaked at 30 days, indicating heightened osteogenic activity and extracellular matrix remodelling. By 60 days, a slight decline was observed; however, levels remained significantly elevated compared to baseline, suggesting continued bone adaptation in response to functional stimulation.

Conclusion

Myofunctional therapy with a Twin Block appliance elicits a measurable biological response indicative of bone remodelling in adolescents with skeletal Class II malocclusion. The increase in salivary levels of key osteogenic markers validates the efficacy of functional appliance therapy and highlights the utility of saliva as a non-invasive diagnostic tool for monitoring treatment-induced bone changes.

## Introduction

Class II malocclusion is frequently associated with one or more of the following conditions: mandibular retrognathism, anterior displacement of the maxilla, increased vertical dimension of the posterior maxilla, mandibular fossa in a posterior position, narrow maxilla, and a combination of these factors. In Class II skeletal malocclusion, mandibular retrognathism seems to be the major contributing factor [1]. A patient is considered retrognathic if there is inadequate mandibular growth, particularly in the anteroposterior (AP) plane [2]. Intricate structural design changes in genetic and environmental factors may have a significant impact on mandibular development. Distortion of the maxillary and/or mandibular development is skeletal malocclusion, which has a significant effect on the location, alignment, and health of the primary and permanent teeth. With an incidence of 1/1,500 live births, micrognathia, a small mandible or maxilla, is the most prevalent cause of skeletal malocclusion [3]. Mandible retrusion prevalence runs from 52% to 56% in the general population [4].

The most typical therapy for mandibular retrusion is condyle traction, which involves moving the mandible forward. Mandibular protrusion stimulates the condyle more effectively, causing sagittal growth [5]. Despite being classified as articular cartilage, mandibular condylar cartilage exhibits several distinct biological characteristics, including its embryonic origin, ontogenetic development, postnatal growth pattern, and histological structure. The capacity for adaptive remodelling in response to external stimuli during or after normal growth is what distinguishes condylar cartilage most from other types of cartilage. The underlying justification for orthodontic functional therapy is the adaptation of condylar cartilage to mandibular forward orientation, which corrects jaw discrepancies by modifying mandibular development. The biomolecular route, which starts with chondrogenesis and ends with osteogenesis, is used to carry out the adaptive remodelling of condylar cartilage. Chondrogenesis is triggered during condylar adaptation when external stimuli, such as condylar shifting, cause mesenchymal cells in the cartilage's articular layer to differentiate into chondrocytes, which multiply and eventually mature into hypertrophic cells. During adaptive remodelling, the expression of regulatory growth factors, which direct and regulate chondrocyte phenotypic conversions during chondrogenesis, rises to facilitate the transition from chondrogenesis to osteogenesis, a process in which hypertrophic chondrocytes and matrices degrade and are replaced by bone. Increased neovascularisation also contributes to the shift by sending in osteoblasts, which eventually lead to the development of new bone underlying the degraded cartilage [6].

The markers for each stage of condylar growth must be determined, and the impact of functional stimulation on the growth pattern must be assessed. However, the effects of functional appliance therapy on the correction of Class II malocclusions remain controversial. Some studies have shown a positive growth response to condyle traction, while others have shown a negative or no response to mandibular advancement [7]. Nearly all of the works have been attempting to learn more about chondrogenesis; therefore, this study concentrated on the subsequent step to determine whether functional stimulation of the mandible would cause the condyle osteoblasts to express various bone-forming markers. Three particular bone markers are examined in this study.

The initial preference will be for collagen type I, as its synthesis is closely regulated and it is expressed throughout the entire process of bone formation. It maintains the structural integrity of bone, and a deficiency of type I collagen causes severe disorders. Other well-known specific markers for osteogenesis are osteocalcin and osteonectin, which are non-collagenous proteins. In comparison to many other bone proteins, osteocalcin has a low molecular weight (approximately 6 daltons), which may not necessitate substantial cleavage to separate it from the bone matrix [8]. Additionally, osteocalcin is a regulator of mechanotransduction and is solely produced by osteoblasts. Osteonectin is of particular interest as it plays a vital role in bone mineralisation and is now emerging as a promising area of research for the prevention and treatment of bone-related problems. Diagnostic biofluids (saliva and blood serum) contain similar proteins and RNA, so the diagnostic medium in this study will be saliva instead of blood serum because it is more easily collected and less invasive than serum, which makes the procedure more acceptable to patients and more conducive to a stress-free appointment. The functional stimulation is increased by condyle traction, and it significantly interferes with the expression of type 1 collagen, osteonectin, and osteocalcin. It opens a new framework for therapeutic strategies that address temporomandibular joint issues as well as mandible retrusion [9].

## Materials and methods

Source of data

A total of 10 subjects were selected from patients who reported to the Department of Orthodontics and Dentofacial Orthopaedics, Mathrusri Ramabai Ambedkar Dental College and Hospital, Bengaluru, and met the inclusion and exclusion criteria. Prior to inclusion in the study, written informed consent was obtained from all participants or their legal guardians, in accordance with ethical research standards. The study protocol was reviewed and approved by the Institutional Ethics Committee of M.R. Ambedkar Dental College and Hospital, Bengaluru.

Inclusion criteria

The study included healthy individuals with no history of systemic diseases within the age range of 8-15 years, ensuring that all participants were in the active growth phase. Skeletal Class II malocclusion due to mandibular retrognathism was confirmed through composite cephalometric analysis. To standardise the sample and minimise variability, all orthodontic appliances were fabricated by the same technician in the Department of Orthodontics laboratory. Participants and their parents were thoroughly informed about the study protocol, and written informed consent was obtained prior to the commencement of treatment. Strict compliance with oral hygiene instructions provided by the investigator was required throughout the treatment duration. Mandibular advancement was carried out following the "Rule of 10" protocol [10], and the construction bite was recorded in modelling wax at a position at least 3 mm posterior to the most protrusive mandibular position [10], to ensure effective forward positioning and optimal treatment response.

Exclusion criteria

Participants were excluded from the study if there was a history of developmental disabilities, including learning disabilities, attention deficits, hearing impairments, or speech abnormalities, as these may indicate an underlying syndromic genetic disorder. A positive family history of genetic disorders was also considered a criterion for exclusion. Additional exclusion factors included a history of maternal illness during pregnancy and parental exposure to medications such as anticonvulsants or retinoic acid derivatives, as well as tobacco or alcohol consumption during pregnancy. Patients with any known systemic or endocrine disorders were not included. Furthermore, individuals who were deemed non-compliant with treatment or oral hygiene instructions were excluded from the study to ensure consistency and reliability of outcomes.

Methodology

This prospective study, conducted from August 23, 2023, to June 20, 2025, involved screening 10 patients scheduled to undergo myofunctional therapy at the Department of Orthodontics and Dentofacial Orthopaedics, M.R. Ambedkar Dental College and Hospital, Bengaluru, based on predefined inclusion and exclusion criteria. Prior to participation, all patients provided written informed consent in accordance with institutional ethical guidelines. Pre-treatment lateral cephalograms were obtained for each participant and subsequently traced for analysis. To minimise inter-examiner variability, cephalometric landmarks were identified independently by two experienced orthodontists. Skeletal malocclusion was assessed using a composite cephalometric analysis. All cephalometric measurements were performed digitally using the NemoCeph software (Nemotec, Madrid, Spain) to minimise measurement bias and ensure greater accuracy.

Saliva collection

Unstimulated saliva samples were collected from all participants during their morning appointments to minimise diurnal variation in salivary biomarkers. Participants were instructed to refrain from eating or drinking for at least one hour prior to sample collection. Participants were asked to tilt their heads slightly forward and passively drool into sterile, safety-locked 2 mL Eppendorf tubes. Care was taken to ensure that no food debris or blood contaminated the samples.

Samples were collected at four time points: baseline, immediately prior to the placement of the orthodontic appliance, and on Days 15, 30, and 60 following the initiation of myofunctional therapy. All samples were properly labelled and stored under appropriate conditions for subsequent biochemical analysis.

Storage

Following collection, each saliva sample was mixed with an appropriate buffer solution to preserve protein integrity during storage. The buffered samples were stored at -82 °C in a deep freezer (Model 5991, Thermo Fisher Scientific, Waltham, MA, USA), ensuring optimal conditions for biomarker stability. Samples were stored under these conditions for up to 280-380 days without significant degradation of the analytes. Prior to biochemical analysis (ELISA, enzyme-linked immunosorbent assay), the samples were transported to the laboratory in a thermo-sealed container containing ice packs to maintain the cold chain and prevent protein denaturation. All samples were handled in accordance with standard biosafety and transport protocols.

Removable myofunctional appliance: Twin Block

Skeletal Class II growing patients treated with Clark's Twin Block appliance were included in this study [11]. A good set of dental impressions was required for the fabrication of the appliance. The construction bite was recorded using modelling wax, ensuring proper mandibular positioning. The appliance design included key components such as a labial bow, delta clasp, ball-end clasp, and a cold-cure acrylic baseplate.

Mandibular advancement was performed according to the "Rule of 10" protocol, which provides a standardised approach for functional appliance therapy. The construction bite was taken at a position at least 3 mm posterior to the patient's most protrusive mandibular position to ensure effective forward positioning and functional adaptation.

Instructions

Participants were instructed and encouraged to wear the myofunctional appliance consistently throughout the study period. They were advised to keep the appliance in the mouth at all times, except during oral hygiene practices such as brushing and flossing. During the initial adaptation period, patients were permitted to remove the appliance while eating. However, they were gradually encouraged to learn to eat with the appliance in place to maximise functional adaptation and therapeutic effectiveness. Patient compliance and appliance usage were assessed and reinforced at each follow-up visit. Progress was monitored at every appointment to ensure proper use and to make any necessary adjustments.

Statistical analysis

To compute the required sample size a priori for a one-tailed test, key inputs include an effect size (dz) of 0.80, an alpha error probability (α) of 0.10, and a desired power (1-β) of 0.85. From these inputs, outputs such as the noncentrality parameter (δ) of 2.5298221, critical t value of 1.3830287, and degrees of freedom (Df) of 9 are derived. The total sample size needed is 10, resulting in an actual power of 0.8702413, ensuring the study is adequately powered to detect effects.

The sample size estimation was performed at 10% alpha error (α = 0.10), with an effect size of 80% (based on Cohen's large effect size for the mean difference in the expression of type I collagen, osteocalcin, and osteonectin between before and after the myofunctional therapy period) and the power of the study at 85%, revealing that a minimum of 10 samples was necessary for the present study. Therefore, each study group consisted of 10 patients with retrognathic mandibular conditions. IBM SPSS Statistics for Windows, Version 22 (Released 2013; IBM Corp., Armonk, New York), was used to perform statistical analyses.

Descriptive statistics

A descriptive analysis of all explanatory and outcome parameters was conducted using means and standard deviations for quantitative variables and frequencies and proportions for categorical variables. For inferential statistics, the Student's paired t-test or Wilcoxon signed-rank test was used to compare the mean levels of type I collagen, osteocalcin, and osteonectin before and after the myofunctional therapy period. An independent Student's t-test/Mann-Whitney test was used to compare the mean levels of type I collagen, osteocalcin, and osteonectin between male and female patients before and after the myofunctional therapy period. The level of significance will be set at P<0.05.

## Results

The study population consisted of 10 adolescent participants, with ages ranging from 11 to 14 years and a mean age of 12.70 ± 1.25 years. The age distribution was relatively uniform, with 30.0% (n = 3) of the subjects aged 11 years, 40.0% (n = 4) aged 13 years, and another 30.0% (n = 3) aged 14 years. This reflects a well-distributed age representation within the early adolescent period, ensuring age-related variations in treatment response are minimised. Regarding gender, the cohort consisted of 60.0% males (n = 6) and 40.0% females (n = 4), indicating a modest predominance of male participants. Although the sample size is limited, this gender distribution provides a reasonable basis for preliminary observations across both sexes (Table 1).

**Table 1:** Age and gender distribution among study subjects

Variable	Category	n	%
Age	11 yrs.	3	30.0%
	13 yrs.	4	40.0%
	14 yrs.	3	30.0%
	-	Mean	SD
	Mean	12.70	1.25
	Range	11–14 yrs.	
Gender	Males	6	60.0%
	Females	4	40.0%

The levels of collagen type I (ng/mL) increased significantly over time following the initiation of myofunctional therapy. At baseline, the mean concentration was recorded at 95.014 ± 9.013 ng/mL. By 15 days, there was a noticeable increase, with a mean of 117.412 ± 7.598 ng/mL. At 30 days, collagen type I levels peaked at a mean of 164.324 ± 10.655 ng/mL. However, by 60 days, a decrease was observed, with the mean concentration falling to 141.504 ± 11.572 ng/mL. The statistical analysis revealed a significant variation in collagen type I levels over the measured intervals (p < 0.001), indicating a pronounced response to myofunctional therapy over time, with an initial rise followed by a decline at a later stage (Table 2).

**Table 2:** Comparison of mean values of different study parameters between different time intervals using the repeated measures ANOVA test * Statistically significant

Parameter	Time intervals	N	Mean	SD	Min	Max	p-value
Collagen Type I (ng/mL)	Baseline	10	95.014	9.013	85.60	110.81	<0.001*
	15 Days	10	117.412	7.598	106.20	126.56	
	30 Days	10	164.324	10.655	147.22	180.22	
	60 Days	10	141.504	11.572	124.93	160.79	
Osteocalcin (ng/mL)	Baseline	10	19.089	5.556	13.64	29.40	<0.001*
	15 Days	10	41.127	5.163	33.70	48.79	
	30 Days	10	79.590	6.711	71.27	87.26	
	60 Days	10	61.231	5.767	53.73	70.79	
Osteonectin (ng/mL)	Baseline	10	33.723	3.576	27.99	40.57	<0.001*
	15 Days	10	45.813	3.254	41.24	50.85	
	30 Days	10	71.917	3.439	67.10	78.53	
	60 Days	10	56.472	4.497	50.43	65.84	

The levels of osteocalcin (ng/mL) also demonstrated a significant variation with time. The mean concentration was 19.089 ± 5.556 at baseline. After 15 days, the levels increased markedly to a mean of 41.127 ± 5.163. At 30 days, the levels reached a peak with a mean of 79.590 ± 6.711. Subsequently, a decline was observed at 60 days, with the mean decreasing to 61.231 ± 5.767. The significant p-value (<0.001) indicated that osteocalcin levels changed considerably over the specified time points, reflecting a dynamic biological response to therapy, with a rapid increase followed by a partial reduction.

Similar to the other parameters, osteonectin (ng/mL) exhibited significant fluctuations across the time intervals evaluated. The mean level at baseline was 33.723 ± 3.576. It increased progressively to 45.813 ± 3.254 at 15 days. The highest mean level was recorded at 30 days, at 71.917 ± 3.439. Following this peak, the levels declined to 56.472 ± 4.497 at 60 days. The statistical analysis confirmed that these changes over time were significant (p < 0.001), indicating a notable biological response to the therapy, characterised by an initial rise followed by a subsequent decrease.

For collagen type I, the mean difference between baseline and 15 days was -22.40, which was statistically significant (p = 0.001), indicating a decrease in levels at 15 days (Table 3). The difference between the baseline and 30-day values was -69.31 (p < 0.001), indicating a substantial decline at this time point. Comparatively, the change between baseline and 60 days was -46.49 (p < 0.001), reflecting a significant reduction over this period. The difference between 15 days and 30 days was -46.91 (p < 0.001), illustrating a significant decrease, while the difference between 15 days and 60 days was -24.09 (p = 0.001), indicating continued reduction, and between 30 days and 60 days was 22.82 (p < 0.001), demonstrating a significant increase from 60 days to 30 days.

**Table 3:** Multiple comparisons of mean differences in different study parameters between different time intervals using Bonferroni's post hoc test * Statistically significant BL: baseline

Parameters	Values	BL vs. 15D	BL vs. 30D	BL vs. 60D	15D vs. 30D	15D vs. 60D	30D vs. 60D
Collagen Type I	Mean Diff	-22.40	-69.31	-46.49	-46.91	-24.09	22.82
	p-value	0.001*	<0.001*	<0.001*	<0.001*	0.001*	<0.001*
Osteocalcin	Mean Diff	-22.04	-60.50	-42.14	-38.46	-20.10	18.36
	p-value	<0.001*	<0.001*	<0.001*	<0.001*	<0.001*	<0.001*
Osteonectin	Mean Diff	-12.09	-38.19	-22.75	-26.10	-10.66	15.45
	p-value	<0.001*	<0.001*	<0.001*	<0.001*	0.001*	<0.001*

The mean difference in osteocalcin between baseline and 15 days was -22.04, which was statistically significant (p < 0.001). The difference between baseline and 30 days was -60.50 (p < 0.001), indicating a large decline, while the change between baseline and 60 days was -42.14 (p < 0.001). The difference between 15 days and 30 days was -38.46 (p < 0.001), and between 15 days and 60 days was -20.10 (p < 0.001), both indicating significant reductions. Additionally, the comparison between 30 days and 60 days revealed a significant difference of 18.36 (p < 0.001), reflecting a notable increase.

For osteonectin, the difference between baseline and 15 days was -12.09 (p < 0.001), indicating a significant decrease. The difference between the baseline and 30-day values was -38.19 (p < 0.001), also indicating a significant reduction. Similarly, the change between baseline and 60 days was -22.75 (p < 0.001). The difference between 15 days and 30 days was -26.10 (p < 0.001), and between 15 days and 60 days was -10.66 (p = 0.001), both indicating significant decreases. The comparison between 30 days and 60 days had a difference of 15.45 with a p-value less than 0.001, showing a significant increase in levels at 60 days compared to 30 days.

Summary

The results indicated that myofunctional therapy led to significant biochemical changes in the study parameters over the observed time intervals. Specifically, levels of collagen type I, osteocalcin, and osteonectin increased notably from baseline, reaching their highest levels around 30 days, which suggested an active anabolic or tissue regeneration response during this period. Following this peak, a decline was observed at 60 days, although the levels remained elevated compared to the initial baseline, indicating ongoing biological activity, albeit with a modulation or stabilisation phase. Multiple pairwise comparisons revealed significant differences between all time points, confirming these dynamic fluctuations. These findings demonstrated that the therapy prompted a progressive response in tissue-related biomarkers, reflecting phases of tissue formation and remodelling, which are critical for understanding the biological impact and timing of therapeutic effects over the course of treatment.

## Discussion

This study aimed to evaluate the systemic biochemical response to Twin Block therapy in adults with skeletal Class II malocclusion by assessing serum levels of three key bone and extracellular matrix (ECM) biomarkers: collagen type I, osteocalcin, and osteonectin. At baseline, 15 days, 30 days, and 60 days after the start of therapy, the periodic expression of these markers was monitored. All of the biomarkers displayed a similar trend in the results: a notable upregulation that peaked at 30 days and then moderately declined at 60 days, yet remained higher than the baseline.

Functional appliances are integral to the early treatment of skeletal Class II malocclusions, primarily by facilitating forward mandibular displacement through neuromuscular modulation and skeletal-dentoalveolar adaptation [12,13]. Among the commonly used appliances, both monoblock and Twin Block designs have been shown to effectively stimulate mandibular growth. However, comparative studies reveal that the Twin Block appliance, when worn full-time, produces more significant vertical skeletal changes, including increased mandibular plane and gonial angles, and a marked reduction in overbite, suggesting enhanced vertical skeletal remodelling [14].

Collagen type I, a primary structural protein, is typically involved in early matrix synthesis and osteogenesis [15]. Osteonectin (also known as SPARC or secreted protein acidic and rich in cysteine) facilitates the organisation of collagen fibres and promotes mineral deposition. It is a multifunctional glycoprotein that bridges early matrix formation with subsequent mineralisation. In our results, osteonectin levels increased from 33.723 ng/mL at baseline to 71.917 ng/mL at 30 days, then modestly declined to 56.472 ng/mL at 60 days. This pattern is in line with osteonectin's dual-phase role, where it initially promotes collagen binding and matrix organisation, followed by facilitating hydroxyapatite nucleation and mineral deposition. Its continued elevation even after 60 days suggests that bone stabilisation and ECM maturation are still actively ongoing. Although osteonectin is less frequently discussed in the orthodontic context, animal studies on mandibular advancement have shown that osteonectin gene expression increases in craniofacial bone, particularly in the condylar region. This supports our interpretation that Twin Block therapy influences not just alveolar bone but also the basal and condylar components of the craniofacial skeleton [16]. Osteocalcin, produced by mature osteoblasts, is a recognised marker of bone turnover and active mineralisation during later stages of remodelling [17]. In our study, all three biomarkers peaked at 30 days and remained elevated at 60 days compared to baseline, suggesting a temporally coordinated biological response to functional orthopaedic loading.

Collagen type I, the predominant structural protein in bone ECM, is essential for mechanical strength and osteogenesis. It also serves as an early biochemical indicator of the bone's response to mechanical stress. In our study, collagen levels increased from 95.014 ng/mL at baseline to a peak of 164.324 ng/mL at 30 days, then tapered to 141.504 ng/mL at 60 days. This sustained elevation suggests active collagen synthesis and ECM remodelling during appliance therapy. Previous animal model studies have demonstrated that mandibular advancement can upregulate collagen-related genes, such as *COL1A1*, even in adult bone tissue [18].

Osteocalcin levels increased from 19.089 ng/mL at baseline to 79.590 ng/mL at 30 days, then decreased slightly to 61.231 ng/mL by day 60. The timing and magnitude of this increase reflect a robust osteogenic response to the Twin Block appliance. The persistent elevation at day 60 suggests continued mineralisation and remodelling, highlighting the skeletal plasticity that persists in late adolescence. This finding is consistent with prior literature, which reports elevations in osteocalcin during orthodontic tooth movement, peaking within the first two to three weeks [19]. However, the delayed peak at 30 days in our adolescent cohort may reflect reduced osteoblastic efficiency and a slower remodelling pace due to hormonal, vascular, and cellular differences that emerge with age. Nevertheless, the sustained elevation of osteocalcin at 60 days reinforces the long-term remodelling capability of adolescent bone in response to sustained functional loading. This also suggests that even in the later stages of adolescent development, there is sufficient plasticity in skeletal tissues to allow for effective orthopaedic correction.

According to several studies, appropriate condylar growth depends on the lateral pterygoid muscle's activity. Increased condylar development cannot be stimulated without pterygoid muscle activation. The "lateral pterygoid hypothesis" is the term used to describe this. Following the use of functional appliances, rats and monkeys have shown increased lateral pterygoid muscle activity, linked to increased condylar cartilage growth. However, due to differences in the temporomandibular regions, such as the muscles and joints, it is not feasible to directly apply the findings from animals to humans [20].

In the experimental study conducted by Rabie et al. on condylar adaptations following mandibular advancement in rats, the results are as follows. In the posterior part of the condyles, endochondral ossification was typical. On the third day of the experiment, no changes were observed. Around the area connected to the retrodiscal pad, new cartilage started to form on day seven. Type X collagen may be immunolocalised later in the hypertrophic layer, while type II collagen expression rose. On day 21, the highest levels of types II and X collagen expression were observed. The corresponding increases were 248% and 540%. Day 30 of advancement showed the highest amount of new bone development, with a 319% increase in new bone formation. On experimental day 60, new bone growth was still greater than that of the controls, despite a decrease from experimental day 30 onward. Our study's results align with those of Rabie et al., as all biomarkers in our study displayed a similar trend: a notable upregulation that peaked at 30 days, followed by a moderate decline at 60 days, while still remaining higher than baseline [21].

The biomarkers in our investigation exhibited significant overexpression, which was higher than baseline, peaked at 30 days, and subsequently decreased moderately at 60 days. The decline in biomarker levels may be attributed to dental compensation, which manifests as the forward movement of the lower teeth. This movement can cause the mandibular base to shift backwards, thereby explaining the decrease in new bone development. Therefore, a decrease in osteogenesis in the glenoid fossa results from a reduction of the disc ligament's elastic stretch and, consequently, less pull on the periosteum. On the other hand, the decrease in new bone production observed can be partially explained by the differentiation of resident osteoprogenitor cells into bone cells, which in turn reduces the number of mesenchymal cells [22].

Limitation

This study is not without limitations. The small sample size (n = 10) limits the statistical robustness of the findings and reduces their generalisability to the broader population. A larger cohort would be necessary to validate these observations and enhance the reliability of the results. Additionally, the absence of a control group, such as untreated adults or those undergoing alternative interventions, restricts the ability to draw definitive causal inferences about the specific effects of Clark's Twin Block therapy on biomarker modulation. Another important limitation is the exclusive use of saliva for biomarker analysis. While saliva provides a systemic overview of bone metabolic activity, it may not accurately capture localised changes occurring within the alveolar bone and surrounding periodontal structures.

Future research incorporating site-specific matrices may offer a more precise understanding of the localised biological response to myofunctional therapy and evaluating it, especially in adult populations where skeletal adaptability is reduced but not absent.

## Conclusions

This study shows that myofunctional therapy with Clark's Twin Block appliance not only repositions the jaw but also stimulates molecular-level bone remodelling in skeletal Class II malocclusion. A 60-day rise in salivary biomarkers (collagen type I, osteocalcin, and osteonectin) reflects active bone formation aligned with mandibular advancement. The findings highlight that functional appliances evoke complex biological responses, not just mechanical changes, paving the way for personalised orthodontic care. Salivary biomarker monitoring offers a non-invasive, real-time method to guide treatment adjustments and predict outcomes more accurately.

Sustained biomarker activity, especially in adolescents and adults, suggests that remodelling continues beyond early therapy, supporting the need for individualised treatment durations. This research bridges the fields of orthodontics and molecular biology, promoting biomarker-guided, evidence-based therapy for more efficient, comfortable, and lasting correction of Class II malocclusion.
